# Traumatic episodes experienced during the genocide period in Rwanda influence life circumstances in young men and women 17 years later

**DOI:** 10.1186/1471-2458-13-1235

**Published:** 2013-12-28

**Authors:** Lawrence Rugema, Ingrid Mogren, Joseph Ntaganira, Krantz Gunilla

**Affiliations:** 1 Department of Community Health, School of Public health, National University of Rwanda, Kigali, Rwanda; 2 Department of Clinical Sciences, Obstetrics and Gynecology, Umeå University, Umeå, Sweden; 3 Department of Public Health and Community Medicine, The Sahlgrenska Academy at Gothenburg University, Gothenburg, Sweden

**Keywords:** Traumatic episodes, Genocide, Long-term effects, Young adults, Harvard Trauma Questionnaire, Rwanda

## Abstract

**Background:**

During Rwanda’s genocide period in 1994, about 800,000 people were killed. People were murdered, raped and seriously injured. This retrospective study investigated prevalence and frequency of traumatic episodes and associated psychosocial effects in young adults in Rwanda over the lifetime, during the genocide period and in the past three years.

**Methods:**

This is a cross-sectional population-based study conducted among men and women, aged 20 to 35 years, residing in the Southern province of Rwanda. The study population, randomly selected in a multi stage procedure, included 477 females and 440 males. Data collection was performed through individual interviewing with a structured questionnaire during the period December 2011- January 2012. The Harvard Trauma Questionnaire was used to assess traumatic episodes. All data was sex-disaggregated. Differences between groups were measured by chi square and Fischer’s exact test. Associations with socio-demographic and psychosocial factors were estimated by use of odds ratios with 95% confidence intervals in bi- and multivariate analyses.

**Results:**

The participants in this study were 3 to 18 years of age in 1994, the year of the genocide. Our sample size was 917 participants, 440 men and 477 women. Women were to a higher extent exposed to traumatic episodes than men during their lifetime, 83.6% (n = 399) and 73.4% (n = 323), respectively. During the genocide period, 37.5% of the men/boys and 35.4% of the women/girls reported such episodes while in the past three years (2009-2011) 25.0% of the men and 23.1% of the women did. Women were more exposed to episodes related to physical and sexual violence, while men were exposed to imprisonment, kidnapping and mass killings. Victims of such violence during the genocide period were 17 years later less educated although married (men OR 1.47; 0.98-2.19; women OR 1.54; 1.03-2.30), without children (men OR 1.59; 1.08-2.36; women OR 1.86; 1.11-3.08) and living under extremely poor circumstances.

**Conclusion:**

The participants in this population-based study witnessed or experienced serious traumatic episodes during the genocide, which influenced their life circumstances 17 years later. Such traumatic episodes are however still taking place. The reasons for this need further investigation.

## Background

Extermination of a large part of the Tutsi minority ethnic group by the Hutu extremists is what characterized Rwanda during the spring of 1994, where in a three months’ period at least 800,000 people were massacred primarily by other civilians [1-3].

Most people were killed in their local communities by perpetrators who were known to the victims. Women were systematically raped and some later hatched to death, and children were also victims of such violence. Tens of thousands of women were intentionally infected with HIV, sexually humiliated and mutilated by the attackers [4-7]. Two million people fled their homes and became refugees in neighbouring countries [8]. It tore Rwandans apart despite having a common history and speaking the same language and now remains as a difficult past [9].

The immediate effects of the genocide on children were investigated in a study conducted in 1995, among 30 primary and secondary schools in various communes in Rwanda. Over 90% of the respondents witnessed killings and their lives were threatened, one third lost closer family members or witnessed rape as well as mutilation and 15% hid under dead bodies for protection [10]. Another study, conducted 13 months after the genocide, revealed inability of many of the young victims to concentrate due to what they had witnessed [11].

The government of Rwanda established a system of community-based conflict resolution courts in 2005, known as Gacaca. These community courts were put in place to locally try thousands of genocide related suspects for having taken part in the massacres [12-14]. The Gacaca hearings allowed community members to participate as witnesses. The month of April is the Genocide Memorial Period when a number of commemoration gatherings are held and testimonies given of what survivors went through, resulting in rewinding of psychological trauma and potential reprisals [15].

Although the legacy of the genocide has become part of life for Rwandans, including those who were children at the time, Rwanda has developed into a society with economic growth and social development, and where community structures allow collective civic engagement to ensure good governance and social protection of the population [16].

Enrolment in basic primary education is currently about 96% with a somewhat higher enrolment for girls than for boys (97.5 and 94.3%). These figures are the highest ever in the history of the country, but with a dropout rate of 11.4%, i.e. children do not finish primary education [17]. Currently 77% of adult females and 82% of adult males are considered literate in Rwanda [18].

The post genocide Rwanda is characterized by women’s empowerment. In 1999, property laws were amended and since then women have the right to inherit land and other valuable assets [19]. The Rwanda constitution legislates that at least 30% of senators should be women [20]. As a consequence, several leadership positions are occupied by women. Currently, 56% of the parliamentarians are women, which positions Rwanda’s parliament as the world’s leading country in this respect [21].

In this particular study, the prevalence and frequency of traumatic episodes experienced by young adults, 20-35 years of age, were explored during lifetime, the genocide period and in the past three years. Possible associations with psychosocial factors were further investigated for long-term effects. This study is part of a larger project on violence and traumatic episodes, mental health and barriers to care, The Rwandan Violence, Mental Health and Barriers to Care project (RwVMHBC- project).

## Methods

This cross-sectional population-based study took place in the Southern province of Rwanda and included adult men and women, aged 20–35 years. This region is mainly rural but includes also an urban city, Butare. As mental health was the overall RwVMHBC project outcome, the sample size was calculated based on the prevalence of depression (20%) in men and women in Rwanda [22]. To detect a 1.5 fold risk increase of depression, with 80% probability, the sample size was estimated to 815 people after taking non-responders into consideration. As the prevalence of depression in men is generally lower than in women [22], it was decided to increase the sample size to 900. A total population of 917 individuals was finally included in the study, 440 (48%) men and 477 (52%) women. The data was collected in the period December 2011 to January 2012.

### Data collection

A two-stage random selection of participants was done by use of the nation-wide demographic health survey procedure to identify households for inclusion in the survey. Rwanda is divided into four provinces and the Southern Province with an estimated population of 2.226.000 was chosen as it includes rural and urban areas. It is divided into eight districts. A complete list of all villages and households was made available from the National Institute of Statistics of Rwanda (NISR).

Out of the total number of 3512 villages in the eight districts, 35 Primary Sampling Units (villages) were selected, representing 10% of the total number of villages in the province. In each district, the number of villages selected was proportional to the total number of villages, using Epi-Info to generate random numbers. The number of households for inclusion in each village was then proportional to the total number of households in each selected village.

One young adult was selected from the first household closest to the centre of the village. If the first eligible participant was a female, the next eligible participant was to be a male. Only one interview was conducted with an eligible individual in each household for ethical and security reasons. If there was no eligible person living in the household, the closest household was approached. The rationale behind choosing a neighbouring household was that living conditions would possibly be similar to that of the primary selected household. If the eligible individual was not at home, the interviewer returned later on, up to three times on different days to interview the selected person. The final sample consisted of 917 participants, 440 men and 477 women, aged 20–35 years, all permanent residents of Rwanda at present. Only two people refused participation in the study, and the final response rate was 99.8%.

A questionnaire was developed based on previously validated instruments. It contained items on traumatic episodes, physical and mental health and access barriers to care, and was translated into Kinyarwanda and back translated by a professional language translator.

Traumatic episodes were measured by a revised version of the Harvard Trauma Questionnaire, an instrument that has been used in several other post conflict settings and validated in different populations and languages [23,24]. The authors state that with careful adaptation to a cultural setting, the scale can be used to assess trauma in non-western populations [25,26]. Of the 34 items included, directly related to trauma experience, 16 were chosen as appropriate for this study. These items were selected and agreed upon by the Rwandan researchers (JN, LR) in discussions with the principal investigator (GK).

A team of 13 clinical psychologists (seven females and six males), experienced in conducting data collection for the School of Public Health, National University of Rwanda were trained for two days to manage the data collection tools. During the two-days training, the instrument was reviewed. A one day pre-testing exercise was carried out and the questionnaire was revised accordingly. Female participants were interviewed by women, and male participants by men to minimize information concealment. The data collection was supervised by persons with previous experience of similar studies (LR, first author and a team supervisor at School of Public Health).

The data collection was implemented by two teams, each consisting of one supervisor and six to seven interviewers. Each team supervisor was responsible for overseeing the work of the team by identifying the households to be visited, observing interviews, managing questionnaires and for re-interviewing a selected number of participants for reliability purposes. Data was entered by four experienced personnel, who draw their expertise from entering DHS data in Rwanda. The supervisor (first author, LR) crossed-checked on a daily basis the quality of data entered and if there were any anomalies, these were corrected immediately.

### Dependent variables

The items on traumatic episodes (Harvard Trauma Questionnaire) [23] ask about having witnessed or own experience of various traumatic episodes. Response categories are 'yes’ and 'no’ with a follow-up question on age at event. Items were treated individually but for further analyses, a summary index was made for episodes during lifetime, the genocide period and the past three years, dichotomized into having experienced at least one traumatic episode as the exposed, and no such exposure constituted the reference category.

### Independent variables

Socio-demographic and psychosocial variables from the Rwanda Demographic and Health survey 2010, were used with slight adjustments. *Age* was categorized into three groups 20–24, 25 –29 and 30–35 years of age and for further analyses, dichotomized with the youngest age group as the reference category. *Marital status* was classified into married or cohabiting as opposed to being widowed, divorced or single.

*Number of children* was divided into having no children, 1–3 children, and >3 children, later dichotomized into having or not having children with the latter as the reference category. *Educational level* was divided into three groups: secondary school or university level, completed primary school or vocational training, and incomplete primary school; then dichotomized with the highest educational level as the reference category. *Ever been to school* was categorized into 'yes’ and 'no’ with 'yes’ being the reference category*. Employment status* was divided into three groups, i.e. full time paid employment, irregular or seasonal work and no employment. *Personal income per month* was categorized into three groups: >35,000 Rwandan Franc (RWF), 17,000 RWF – 35,000 RWF, and <17500 RWF. For analysis purposes it was dichotomized at the level of 17,500 RWF (equivalent to 30 USD). *Source of income* was divided into four categories: salary, pension, disability grant and no income; in the further analyses any source of income was made the reference category. The *social support measure* consisted of seven items inquiring about support from a relative or a friend when needed (share food, share housing, assisted when ill, borrow money, guidance on how to improve present life situation, support when in personal problems), belonging to any association. A dichotomised variable was created where a 'no’ response to all items was categorized as poor social support and at least one 'yes’ response was categorized as having improved social support.

*Living standard* comprised house type (modern house or shack), toilet facility (flushing or latrine), electricity in the house (yes or no), cooking fuel (paraffin or firewood/dung), and water source (tap water or surface water). The second category of these variables was considered as the negative exposure. A summary variable was constructed where improved standard was equal to having at least one of the improved standards while having none of the improved standards characterised poor living standard.

The *Assets in the household* variable was based on possession of any of the assets (radio, television set, mobile phone, computer, refrigerator, motorcycle, bicycle or car) in the household.

A summary variable was created by adding up the number of assets owned in the household. Not being in possession of any of the assets formed the exposure category.

*Household monthly income* was dichotomised in the same way as personal income, described above.

### Statistical methods

Prevalence was calculated for each specified traumatic episode and a summary variable was constructed for three time periods: lifetime, the genocide period and past three years. The traumatic episodes related to the genocide period (1994) included all cases reported in the period 1994 ± 1 year to take care of recall bias of the exact age of exposure. This procedure was found important as age at episode was inquired about and not the exact timing (year) of each episode. As 17 years have elapsed since the genocide period in 1994 and some of the participants were really young then (from 3 years of age), it is plausible that some were unaware of their exact age, as evidenced in other studies [27]. Traumatic episodes during life time relate to experience of any of the items inquired about during any point in life and past three years included all traumatic episodes experienced in the years 2009–2011.

Data was sex-disaggregated and statistical significance for difference between groups was obtained by use of Pearson’s chi square test and Fischer’s exact test.

Associations between traumatic episodes during lifetime and in 1994 ± 1 year and socio-demographic and psychosocial variables were calculated by use of odds ratios (OR) with 95% confidence intervals (95% CI). Although this is a cross-sectional study, with all data collected at the same point in time, the exposure to traumatic episodes during the genocide period took place about 17 years earlier than measures of current socio-demographic and psychosocial circumstances, which makes it possible to draw cautious conclusions on long-term effects of the trauma. Multivariate logistic regression analyses were also performed, controlling for age, number of children, education and income as statistically significant in the bivariate analyses, or close to, for both men and women. Data were entered into SPSS version 19 (SPSS v 19.0; IBM SPSS Inc.) and this software was used for all statistical calculations.

### Definitions

The variable *traumatic episodes* was here defined as trauma and torture related to mass violence [26].

*Young adults* are defined as men and women aged 20–35 years of age, as is customary in Rwanda.

### Ethical considerations

Permission and ethical clearance was sought from the Rwanda National Ethics Committee. Approval was given with the reference number FWA Assurance No. 00001973, IRB 00001497 of IORG0001100.

Respondents were informed orally and in writing about their possibility to withdraw from the study at any stage. Participants were further informed that anonymity and confidentiality would be kept at all stages of the research project. The structured interviews following the questionnaire were initiated after a written informed consent was granted by the respondents. All interviews were performed in complete privacy, either outside the house or inside, or in a nearby private location, depending on the choice of the study participant. All personal identity information noted in the questionnaires (village, cluster) was never entered into the data analysis tool.

## Results

### Respondents’ characteristics

Of the 440 men and 477 women participating in the study, 80% lived in rural areas and 20% in an urban environment (Table 1). The great majority were generally poor with only few having secondary or university education (men 13.3%, women 17.0%), few were employed and the household income was generally low. Women to a higher extent than men reported not having completed primary school (women 64.4%, men 58.7%). The majority of the households had 1–3 three children and women were generally poorer than men in terms of assets and to a lesser extent earned an income.

**Table 1:** Socio-demographic and psychosocial factors for men and women with p-values for difference between men and women

**Variables**	**Men**		**Women**		**p-value**
***Respondent’s characteristics***	***n***	**%**	***n***	**%**	
**Age groups (N = 908)**					
20-24	148	*33.8*	127	*27.0*	.050
25-29	144	*32.9*	156	*33.2*	
30-35	146	*33.3*	187	*39.8*	
**Marital status (N = 912)**					
Married and cohabiting	236	*53.8*	342	*72.3*	< 0.001
Widowed and divorced	2	*0.5*	33	*7.0*	
Single	201	*45.8*	98	*20.7*	
**Number of children (N = 915)**					
No children	211	*48.1*	96	*20.2*	< 0.001
1-3 children	192	*43.7*	275	*57.8*	
>3 children	36	*8.2*	105	*22.1*	
**Level of education (N = 768)**					
Secondary school or University level	50	*13.3*	67	*17.0*	.006
Complete primary or vocational level	105	*28.0*	73	*18.6*	
Incomplete primary	220	*58.7*	253	*64.4*	
**Ever been to school (N = 915)**					
Yes	369	*84.2*	393	*82.4*	.479
No	69	*15.8*	84	*17.6*	
**Employment status (N = 913)**					
Full time paid employment	55	*12.5*	29	*6.1*	< 0.001
Irregular or seasonal work	33	*7.5*	17	*3.6*	
No employment	348	*79.1*	431	*90.4*	
**Personal income per month (N = 912)**					
More than 35,000 RF	19	*4.3*	11	*2.3*	.005
17,500-35,000 RF	36	*8.2*	19	*4.0*	
Less than 17,500 RF	382	*86.8*	445	*93.3*	
**Source of income (N = 903)**					
Salary	38	*8.7*	9	*1.9*	< 0.001
Pension, disability grant or other	54	*12.3*	34	*7.3*	
No income	347	*79.0*	421	*90.7*	
**Social support (N = 917)**					
Improved	77	*17.5*	63	*13.2*	.081
Poor	363	*82.5*	414	*86.8*	
***Household characteristics***					
**Household monthly income (N = 883)**					
17,500 RF or more	86	*20.5*	103	*22.2*	.566
Less than 17,500 RF	333	*79.5*	361	*77.8*	

N = 917, 440 men and 477 women.

Housing standard was higher for men than for women with more women living in a shack type of housing (Table 2). Access to safe water also indicated a real difference in standard of living between men (77%, n = 338) and women (44%, n = 208). Among household assets, a radio was possessed more than any other item by both women and men (60.1% women, 68.2% men), while bicycles were more often owned by men, however more women than men were in possession of a mobile phone.

**Table 2:** Living standard and Assets in the household, men and women

**Variable**	**Men**		**Women**		**p-value**
**Living standard characteristics**	**n**	**%**	***n***	**%**	
**House type (N = 915)**					
Combined building, flat, modern house, maisonette	189	*43.1*	175	*36.8*	.058
Shack	250	*56.9*	301	*63.2*	
**Toilet facility (N = 910)**					
Flushed, improved latrine	14	*3.2*	10	*2.1*	.071
Latrine or no toilet	422	*95.6*	464	*97.3*	
**Electricity in the household (N = 914)**					
Yes	39	*8.9*	68	*14.3*	.103
No	400	*90.9*	407	*85.3*	
**Cooking fuel (N = 914)**					
Kerosene, paraffin and other fuels	37	*8.4*	41	*8.6*	.896
Firewood and dung	403	*91.6*	433	*90.8*	
**Water source (N = 912)**					
Tap water, borehole water	338	*76.8*	208	*43.6*	<0.001
Surface water	101	*23.0*	265	*55.6*	
***Living standards, summary measure*** ***(N = 917)***					
Improved (at least one of the higher standard items)	366	*83.2*	305	*63.9*	<0,001
Poor (none of the higher standard items)	74	*16.8*	172	*36.1*	
**Assets in the household**					
**Radio (N = 916)**					
Yes	300	*68.2*	286	*60.1*	.011
No	140	*31.8*	190	*39.8*	
**Television (N = 916)**					
Yes	25	*5.7*	30	*6.3*	.781
No	415	*94.3*	446	*93.7*	
**Refrigerator (N = 915)**					
Yes	2	*0.5*	6	*1.3*	.290
No	437	*99.5*	470	*98.7*	
**Bicycle (N = 915)**					
Yes	87	*19.8*	58	*12.2*	.002
No	352	*80.2*	418	*87.8*	
**Motorcycle (915)**					
Yes	4	*0.9*	13	*2.7*	.050
No	435	*99.1*	463	*97.3*	
**Car (N = 915)**					
Yes	3	*0.7*	9	*1.9*	.147
No	436	*99.3*	467	*98.1*	
**Mobile phone (N = 915)**					
Yes	122	*27.8*	160	*33.6*	.062
No	317	*72.2*	316	*66.4*	
**Computer (914)**					
Yes	3	*0.7*	10	*2.1*	.094
No	435	*99.3*	466	*97.9*	
***Assets in household*,** ***summary measure (N = 917*)**					
Improved (At least one of the higher standard items)	323	*73.4*	331	*69.4*	.189
Poor (None of the higher standard items)	117	*26.6*	146	*30.6*	

N = 917, 440 men and 477 women. p-values for difference between men and women.

These data point at the homogeneity of the population and communities in terms of living standard and assets in the households. Most households neither owed a refrigerator nor a car, television or a computer (Table 2).

### Exposure to traumatic episodes

Of the total population, 78.7% had experienced at least one traumatic episode in their life time (Table 3). Women were to a higher extent exposed than men, 83.6% (n = 399) and 73.4% (n = 323), respectively (Table 4). During the genocide period, 37.5% of the men and 35.4% of the women reported such episodes while in the past three years (2009–2011) 25% of the men and 23% of the women reported experience of traumatizing episodes.

**Table 3:** Prevalence of Traumatic events among men and women 20–35 years of age in different time periods

**Traumatic events**	**Life time prevalence (1976–2011)**		**Prevalence in 1994+/-1 yr.^ (genocide period)**			**Prevalence in the past 3 years (2009–2011)**		
	**N**	%*	**N**	%*	%**	**N**	%*	%**
*1. Have you been imprisoned, kidnapped, held captive*	94	10.3	8	0.9	8.7	27	2.9	28.7
*2. Been a refugee, forced to flee from your home to escape danger/persecution*	228	24.9	120	13.1	52.9	9	1.0	3.9
*3. Experienced forced separation from family members*	54	5.9	18	2.0	33.3	3	0.3	5.6
*4. Experienced a life-threatening injury*	133	14.5	29	3.2	21.8	19	2.1	14.3
*5. Experienced a murder or unnatural death of a family member or a friend*	223	24.3	84	9.2	37.7	19	2.1	8.5
*6. Been robbed, mugged, threatened with a weapon*	157	17.1	31	3.4	19.9	30	3.3	19.1
*7. Experienced imprisonment of close family member*	454	49.6	74	8.1	16.3	55	6.0	12.1
*8. Witnessed a traumatic event to a loved one*	306	33.4	41	4.5	13.4	59	6.4	19.3
*9. Ever been raped by a stranger*	22	2.4	2	0.2	8.7	1	0.1	4.5
*10. Ever felt forced to have sex in exchange of money or other benefits*	31	3.4	1	0.1	3.2	5	0.5	16.1
*11. Witnessed repeated violence between family members*	74	8.1	13	1.4	17.6	11	1.2	14.9
*12. Witnessed physical or sexual violence against a family member by someone outside of the family*	54	5.9	9	1.0	16.7	11	1.2	20.4
*13. Witnessed someone being badly injured or killed*	131	14.3	50	5.5	38.5	10	1.1	7.6
*14. Witnessed atrocities, e.g. mass killings mutilated bodies*	161	17.6	100	10.9	61.7	5	0.5	3.1
*15. Been in a combat situation*	114	12.4	32	3.5	27.8	20	2.2	17.5
*16. Other life threatening/disturbing event*	157	17.1	58	6.3	37.4	20	2.2	12.7
*Summary measure of all traumatic events*	722	78.7	334	36.4	46.3	220	24.0	30.5

*Percent of total number of cases.

**Percent of cases of respective act.

^The number of events was calculated from reported age of participant at event and recoded into year 1994.

Total population, N = 917.

**Table 4:** Men and women exposed to various traumatic episodes during life time, in 1994 and in the past 3 years, presented by sex with p-values for sex difference in lifetime estimates

***Traumatic events***	**Men life time**		**Women life time**		**p-values difference lifetime**	**Men 1994 + -1 year**		**Women 1994 + -1 year**		**Men past 3 years**		**Women past 3 years**	
	**N**	**%**	**N**	**%**		**N**	**%**	**N**	**%**	**N**	**%**	**N**	**%**
*1. Imprisoned, kidnapped, held captive*	69	*15.7*	25	*5.2*	< 0.001	3	*0.7*	5	*1.0*	21	*4.8*	6	*1.3*
*2. Been a refugee, forced to flee from home to escape danger/persecution*	96	*21.8*	132	*27.7*	0.055	56	*12.7*	64	*13.4*	4	*0.9*	5	*1.0*
*3. Experienced forced separation from family members*	19	*4.3*	35	*7.3*	0.122	6	*1.4*	12	*2.5*	1	*0.2*	2	*0.4*
*4. Experienced a life-threatening injury*	80	*18.2*	53	*11.1*	0.001	13	*3.0*	16	*3.4*	14	3.2*	5	1.0*
*5. Experienced a murder or unnatural death of a family member or a friend*	99	*22.5*	124	*26.0*	0.248	37	*8.4*	47	*9.9*	10	*2.3*	9	*1.9*
*6. Robbed, mugged, threatened with a weapon*	75	*17.0*	82	*17.2*	0.930	10	*2.3*	21	*4.4*	14	*3.2*	16	*3.4*
*7. Experienced imprisonment of close family member*	206	*46.8*	248	*52.0*	0.129	39	*8.9*	35	*7.3*	26	*5.9*	29	*6.1*
*8. Witnessed a traumatic event to a loved one*	136	*30.9*	170	*35.6*	0.093	17	*3.9*	24	*5.0*	29	*6.6*	30	*6.3*
*9. Ever been raped by a stranger*	5	*1.1*	17	*3.6*	0.011	1	*0.2*	1	*0.2*	0	*0*	1	*0.2*
*10. Ever felt forced to have sex in exchange of money or other benefits?*	6	*1.4*	25	*5.2*	0.001	0	*0.0*	1	*0.2*	3	*0.7*	2	*0.4*
*11. Witnessed repeated violence between family members*	25	*5.7*	49	*10.3*	0.011	7	*1.6*	6	*1.3*	4	*0.9*	7	*1.5*
*12. Witnessed physical/sexual violence against a family member by someone outside of the family*	18	*4.1*	36	*7.5*	0.034	3	*0.7*	6	*1.3*	3	*0.7*	8	*1.7*
*13. Witnessed someone being badly injured or killed*	43	*9.8*	88	*18.4*	< 0.001	14	3.2**	36	7.5**	5	*1.1*	5	*1.0*
*14. Witnessed atrocities, e.g. mass killings, mutilated bodies*	88	*20.0*	73	*15.3*	0.083	62	14.1**	38	8.0**	1	*0.2*	4	*0.8*
*15. Been in a combat situation*	56	*12.7*	58	*12.2*	0.765	16	*3.6*	16	*3.4*	10	*2.3*	10	*2.1*
*16. Any other life threatening or disturbing event*	74	*16.8*	83	*17.4*	0.860	29	*6.6*	29	*6.1*	12	*2.7*	8	*1.7*
*Summary measure of all traumatic events*	323	73*.4*	399	*83.6*	0.132	165	*37.5*	169	*35.4*	110	*25.0*	110	*23.1*

*P < 0.05; **p < 0.005.

N = 917, men 440; women 477.

### Lifetime occurrence of traumatic episodes

Almost half of the study population had experienced imprisonment of a close family member and one third had witnessed a traumatic event to a loved one in their lifetime (Table 1). Being a refugee, forced to flee from home to escape danger/persecution was experienced by 24.9% and about the same proportion had experienced a murder or unnatural death of a family member or a friend (24.3%). Of the total population, 17.6% had to witness atrocities such as mass killings and mutilated bodies.

Gender differences showed that while women were more exposed to traumatic episodes related to physical and sexual violence, such as rape, forced to have sex for benefits, witnessing violence between family members and also to have witnessed sexual violence against a family member by a stranger, men were to a greater extent exposed to imprisonment, kidnapping, mass killings or were badly injured (Table 4). This explains partly what is seen in the Rwandan society today, i.e. a high number of widows after loss of their husbands during the genocide [28].

### The genocide period

Given the circumstances of the 1994 period, some of the traumatic episodes were especially prevalent, as could be expected (Table 1). Being a refugee and forced to flee from home affected 13.1% of the total population but corresponded to 52.9% of the total number of individuals that experienced this particular episode. Further, we found that having to witness atrocities such as mass killings and mutilated bodies mainly happened during this period (10.9% of total population versus 61.7% within this episode). However, in the case of experiencing murder or the unnatural death of a family member or a friend, a significant proportion happened in this period (9.2% versus 37.7%) but it seems the majority of such episodes took place outside of the genocide period. This was also true for imprisonment of a close family member (8.1% versus 16.3%).

More women than men had the experience of witnessing somebody being badly injured or killed, 7.5% compared to 3.2%. Yet, more men (14.1%, n = 62) than women (8.0%, n = 38) had witnessed atrocities such as mass killings and mutilated bodies (Table 4).

### Past three years (2009–2011)

A somewhat worrying finding was that having experienced a murder or unnatural death of a family member still happened in the period 2009–2011. In the past three years, 2.1% of the total population reported such experience, men to a somewhat higher proportion than women. Witnessing physical or sexual violence against a family member was a somewhat more common experience in the past three years than in the genocide period (Table 3). The most frequent exposures in both men and women were however related to imprisonment of a close family member (men 5.9%; women 6.1%) and having witnessed a traumatic episode directed at a loved one (men 6.6%; women 6.3%).

**Figure 1:**
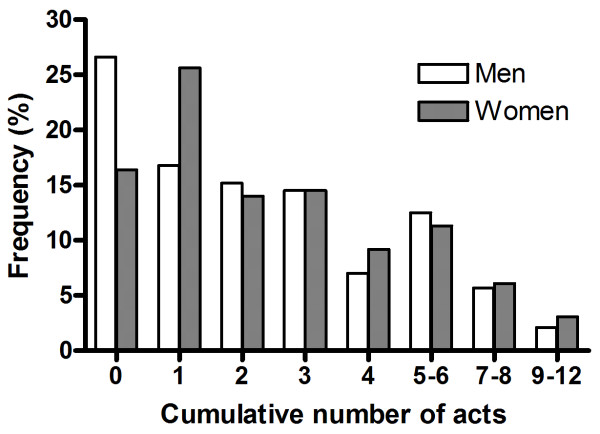
Accumulated number of various traumatic episodes experienced by men and women, life time. N = 440 men and 477 women.

Finally, the accumulated number of the various traumatic episodes during lifetime is displayed in Figure 1. A higher proportion of women had experienced any traumatic exposure. Women also dominated in the highest range, i.e. 7–12 different exposures.

### Traumatic episodes and socio-demographic and psychosocial factors

Associations between traumatic episodes experienced in the genocide period, and socio-demographic and psychosocial factors mirroring current life circumstances were investigated in bi-variate analyses (Table 5). The statistically significant associations hereby illustrate the possible effects of having experienced traumatic episodes about 17 years earlier.

**Table 5:** Associations between traumatic episodes and socio-demographic and psychosocial factors for men and women in the period 1994 ± 1year, presented as crude odds ratios and 95% confidence intervals (Crude OR; CI 95%)

***Variables***	**Men**		**Women**	
***Respondent characteristicS***	**n (%)**	**OR (95% CI)**	**n (%)**	**OR (95% CI)**
**Age groups**				
20 - 29	97 (33.2)	1	94 (33.2)	1
30 - 35	68 (46.6)	1.76 (1.17 – 2.63)	75 (40.1)	1.35 (0.92 – 1.98)
**Marital status**				
Married or cohabiting	95 (40.3)	1	135 (39.5)	1
Single, separated or divorced	69 (34.0)	0.76 (0.52 – 1.13)	34 (26.0)	0.54 (0.34 – 0.84)
**Number of children**				
Have children	97 (42.5)	1	145 (38.2)	1
Have no children	67 (31.8)	1.59 (1.08 – 2.36)	24 (25.0)	1.86 (1.11 – 3.08)
**Ever attended school**				
Attended school	149 (40.4)	1	137 (34.9)	1
Never attended school	15 (21.7)	0.41 (0.22 – 0.76)	32 (38.1)	1.15 (0.70 – 1.88)
**Level of education**				
Completed primary, vocational training, secondary school and university level	68 (43.6)	1	61 (42.4)	1
Incomplete primary & no school	96 (34.5)	1.47 (0.98 – 2.19)	107 (32.3)	1.54 (1.03 – 2.30)
**Employment status**				
Employed	33 (37.5)	1	24 (52.2)	1
Not employed	131 (37.6)	0.99 (0.61 – 1.61)	145 (33.6)	2.15 (1.17 – 3.97)
**Personal Income**				
>17500 (greater than)	139 (36.4)	1	150 (33.7)	1
< 17500 RF (less than)	26 (47.3)	1.56 (0.89 – 2.77)	17 (56.7)	2.57 (1.22 – 5.44)
**Source of income**				
Salary, pension, disability grant or others	33 (35.9)	1	20 (46.5)	1
No income	131 (37.8)	1.08 (0.67 – 1.75)	140 (33.3)	0.57 (0.30 – 1.08)
**Social support**				
Improved	65 (35.5)	1	46 (37.1)	1
Poor	100 (38.9)	1.16 (0.78 – 1.71)	123 (34.8)	0.91 (0.59 – 1.39)
**Household characteristics**				
**Household monthly income**				
Household income > =17500 RF/month	33 (38.4	1	44 (42.7)	1
Household income < 17500 RF	126 (37.8)	0.98 (0.60 – 1.59)	123 (34.1)	0.69 (0.44 – 1.09)
**Living standards**				
Improved; At least one improvement	150 (41.0)	1	106 (34.6)	1
Poor; No improvements out of five	15 (20.3)	0.37 (0.20 – 0.67)	63 (36.6)	1.09 (0.74 – 1.60)
**Assets in the household**				
Improved; at least one of the items	117 (36.2)	1	118 (35.6)	1
Poor; Have none of the items	48 (41.0)	1.23 (0.80 – 1.89)	51 (34.9)	0.97 (0.64 – 1.46)

Men exposed during the genocide period belonged mainly to the higher age group, they were less likely to have children (OR 1.59; 95% CI 1.08-2.36), although married to a woman of about the same age (OR 1.93; 1.12-3.30). The majority had attended school, but our findings also suggest inability to complete primary school, however not statistically significant (OR 1.47; 0.98-2.19). The exposed men were further at a somewhat elevated risk of earning a low income (OR 1.56; 0.89-2.77) than unexposed men, but this was not a statistically significant finding.

Exposed women were more often married but less likely to have any children (OR 1.86; 1.11-3.08), further to be low educated (OR 1.54; 1.03-2.30) and not employed (OR 2.15; 1.17-3.97), with a very low or no income (OR 2.57; 1.22-5.44) but married to someone with complete primary education or higher levels when compared to unexposed women.

These findings for the genocide period were adjusted for in logistic regression analyses controlling for age, number of children, education and income, resulting in minor changes of estimates. Main changes for men were that having no children lost its statistical significance (OR 1.36; 0.85-2.17). For women, only low educational achievement lost its statistical significance (OR 1.46; 0.95-2.24) (not in Table).

For life time exposure to traumatic episodes and socio-demographic and psychosocial factors, no statistically significant associations were found for men (not in Table). For women, there were statistically significant crude associations with not being employed but on a small pension or disability grant, with improved social support, but still with household income being low (not in Table).

## Discussion

This study investigated traumatic episodes among young adults in the Southern province of Rwanda, where life circumstances were found to be similar between households. The majority of the participants were living under somewhat improved housing conditions while about one quarter were in very poor circumstances, with few assets in the household. However, the possession of a mobile phone was the most common asset in a household after a radio and more commonly owned than a refrigerator or a bicycle. In possession of assets, women had less than men, even if more women than men were in possession of a mobile phone, a refrigerator and a computer.

More than one third of the participating men and women recall being exposed to serious traumatic episodes during the genocide but as well during the past three years. Extremely serious exposures, such as being forced to flee the home and to witness mass killings and mutilated bodies mainly occurred during the genocide, with both men and women carrying such experiences.

The violence is to a certain extent still on-going and some of the most commonly occurring episodes in the past three years are of the character that is not expected to occur in peaceful periods, such as being kidnapped or held captive and also violent deaths. The pardoning of perpetrators by the traditional courts (Gacaca) and their subsequent release and return to the community where they lived prior and during the genocide period not only resulted in reconciliation but also unease, fear and even hatred among the survivors, which may explain such on-going violence [29].

Over the lifetime with similar trends during the genocide, women were more exposed to rape, forced sex, and having to witness repeated violence between family members but also to violence exercised by individuals outside of the family. Men were more exposed to mass-killings than women.

Finally, we found indications of how such traumatic episodes may influence future life circumstances as those exposed, both men and women, to a higher extent were low educated, had no children although being married and had low personal incomes compared to those not exposed. For both men and women these effects signal insecurity about the future and psychological distress.

### Findings in relation to other studies

There is a sharp contrast between our findings and those of Neugebauer et. al., as in their study the prevalence for all kinds of traumatic episodes during the genocide is considerably higher, but their study collected its data in 1995 [10]. The respondents were school children aged 8–19 at the time, with fresh memories of the horrors taking place in the previous year. The age span is about the same in the two studies but 16 years have elapsed between the two data collections and the instruments used for obtaining the data were different. The difference in prevalence is most probably also explained by the fact that in our study, the youngest age group was only three to six years when the genocide occurred and these individuals may not have detailed memories of what occurred to be able to report on specific episodes. Those of our respondents that belonged to the higher age groups were more exposed than the younger ones and as well more able to interpret situations and circumstances happening around them. Still, 16 years probably infers some problems with recalling certain events.

Another study investigated violence and trauma exposure about ten years after the genocide among 68 orphans, 13–23 years old [30] that had all been exposed to extreme levels of violence. Older youth (18–23 years) reported having witnessed a massacre more frequently than younger children, and more boys were victims of violent attacks than girls as was also found in our study. A smaller number also reported having experienced such events before and after the genocide, which is similar to our findings.

Finally, in a community-based study from 2010, i.e. performed 16 years after the genocide and one year before our study, exposure to various traumatic episodes were of about the same magnitude as in our study. However, mainly women were included and the sample size was rather small [31].

Not only recall bias including repression may be at hand as years elapse, there is also a risk of inability to remember events from childhood, as described by Felitti and co-workers in their studies on memory disturbances related to *adverse childhood experiences* (ACE). They investigated childhood autobiographical memory disturbance, which is a memory disruption characterized by the inability to remember events from childhood. Acts such as physical, sexual and emotional abuse, separation from parent/s, threatened or hurt by a knife or household member imprisoned were investigated [32]. They found in a population of 9460 men and women that 18% suffered from memory disturbances with a 4.4-fold risk increase for persons with ≥4 ACEs. However, we cannot rule out whether this mechanism was relevant in our study but it might be the case especially for those exposed to accumulated traumatic episodes.

Women were, to higher extent than men, exposed to traumatic episodes in their lifetime, which may be explained by the fact that women more frequently than men are exposed to intimate partner violence over the life course [33]. In support of this assumption, the Rwandan Demographic and Health Survey from 2005 presented data for women’s exposure to partner violence and show that 31% out of 4066 women were exposed to physical violence since the age of 15, and 19% in the past year [34]. No published study is available presenting data on violence exposure in men. However, in another study within this project on intimate partner violence exposure we found that women were considerably more exposed to such violence than men [Umubyeyi A, Mogren I, Ntaganira J, Krantz G: Intimate partner violence directed at young men and women in post-genocide Rwanda: Prevalence and risk factors, Submitted. 2013].

No scientific studies have investigated on-going traumatic episodes in Rwanda. However it is obvious from our study on past three year prevalence that such violence and traumatic episodes are still on-going and should possibly be understood as the aftermath of the genocide. People are in fear when genocide perpetrators return to their villages.

The long-term effects observed in this study are of interest and have not been reported previously. The fact that both men and women were at excess risk of poorer life circumstances and less educational achievements than those unexposed is maybe not of any surprise but demands societal efforts and support as these are young people with many years of productive life remaining. Among most serious outcomes is the childlessness in both men and women. A cautious interpretation is that this may indicate feelings of insecurity about the future accompanied by psychological distress in both men and women.

### Methodological considerations

The selected age groups for this study included children as young as three years at the time of the genocide. This may give rise to underreporting of traumatic exposures among the youngest age group. Recall bias but also memory disturbances and repression may further have troubled our participants, leading to underreporting.

The retrospective nature of the Harvard Trauma Questionnaire, asking about age at episode, potentially predisposes recall bias. The traumatic episodes in the genocide period were therefore calculated as episodes occurring in the year 1994 ± 1 year. This procedure may have caused some over-estimation. Evidence to support our choice of procedure is that the prevalence thus obtained is in line with what has been found in comparable studies [31].

The cross-sectional design allows for investigating long-term psychosocial effects of the traumatic episodes during the genocide period as these happened 17 years before the actual data collection, however, these findings are to be interpreted with some caution as this is not a true longitudinal study and recall bias may be at hand.

The procedure of randomly selecting the participants was done with great care, interviews were performed by trained clinical psychologists, and the non-response rate was negligible. The data collectors were health workers of a small age difference to the participants, which has been shown to improve the accuracy of the reporting in interviews [35]. As Rwanda is a small country with 10 million inhabitants [18], living under rather equal life circumstances, we believe that the findings of this study can be generalised to the entire population.

## Conclusion

Seventeen years after the genocide, young men and women still carry memories of serious adverse experiences during childhood or adolescence. The violence is to a certain extent still on-going and some of the most commonly occurring episodes in the past three years are of the character that is not expected to occur in peaceful periods, such as being kidnapped or held captive and also violent deaths. Gender differences showed that while women were more exposed to traumatic episodes related to physical and sexual violence, men were to a greater extent exposed to imprisonment, kidnapping, mass killings or were badly injured.

Those exposed during the genocide period were also more likely to be childless although married, extremely low-educated and living under poorer circumstances than those not exposed. A cautious interpretation is that this may indicate feelings of insecurity about the future accompanied by psychological distress in both men and women. As this is a young population, societal efforts and support directed at young people with many years remaining of productive life are demanded. Health care services should be made aware of that extreme trauma has extensive long-lasting health effects and access to high quality care is of utmost importance.

## Competing interests

The authors do declare that they have no competing interests whatsoever.

## Authors’ contribution

Gunilla Krantz (GK) designed the project in collaboration with Ingrid Mogren (IM), developed the questionnaire and directed all steps of data collection in collaboration with IM and Joseph Ntaganira (JN). Lawrence Rugema (LR) supervised all the data collection from the field, supervised data entry and cleaning. RL carried out the statistical analyses with support from GK and IM. RL drafted the manuscript, GK, IM and JN read and revised the text until a final version, which was carefully read and accepted by all authors.

## Pre-publication history

The pre-publication history for this paper can be accessed here:

http://www.biomedcentral.com/1471-2458/13/1235/prepub
